# Assessment of activities performed by clinical nurse practitioners and implications for staffing and patient care at primary health care level in South Africa

**DOI:** 10.4102/curationis.v39i1.1479

**Published:** 2016-03-11

**Authors:** Jude Igumbor, Alta Davids, Catharina Nieuwoudt, Jessica Lee, Rifqah Roomaney

**Affiliations:** 1 School of Public Health, University of the Witwatersrand, South Africa; 2 BroadReach Healthcare, South Africa; 3 Department of Health, Western Cape Provincial Government, South Africa; 4 Johns Hopkins University, Baltimore, United States

## Abstract

**Background:**

The shortage of nurses in public healthcare facilities in South Africa is well documented; finding creative solutions to this problem remains a priority.

**Objective:**

This study sought to establish the amount of time that clinical nurse practitioners (CNPs) in one district of the Western Cape spend on clinical services and the implications for staffing and skills mix in order to deliver quality patient care.

**Methods:**

A descriptive cross-sectional study was conducted across 15 purposively selected clinics providing primary health services in 5 sub-districts. The frequency of activities and time CNPs spent on each activity in fixed and mobile clinics were recorded. Time spent on activities and health facility staff profiles were correlated and predictors of the total time spent by CNPs with patients were identified.

**Results:**

The time spent on clinical activities was associated with the number of CNPs in the facilities. CNPs in fixed clinics spent a median time of about 13 minutes with each patient whereas CNPs in mobile clinics spent 3 minutes. Fixed-clinic CNPs also spent more time on their non-core functions than their core functions, more time with patients, and saw fewer patients compared to mobile-clinic CNPs.

**Conclusions:**

The findings give insight into the time CNPs in rural fixed and mobile clinics spend with their patients, and how patient caseload may affect consultation times. Two promising strategies were identified – task shifting and adjustments in health worker deployment – as ways to address staffing and skills mix, which skills mix creates the potential for using healthcare workers fully whilst enhancing the long-term health of these rural communities.

## Introduction

The HIV epidemic has greatly intensified the shortage of human resources for healthcare in sub-Saharan Africa (Callaghan, Ford & Schneider 2010). In 2008, the Development Bank of South Africa (DBSA) estimated that in South Africa alone there is a deficit of at least 79 791 healthcare professionals (HERD 2009). In particular, there is a dire shortage of doctors and nurses in public healthcare facilities in the country (Coovadia *et al.* 2009; Daviaud & Chopra 2008).

In addition to the staff shortage across South Africa, staff members are unevenly distributed between the various facilities, sub-districts and districts (Daviaud & Chopra 2008). This maldistribution is even further exacerbated as a result of the migration of healthcare professionals from rural to urban areas where working conditions may be better (Chen *et al*. 2004). This variation in staffing levels can lead to an inefficient usage of professional staff and possible problems of quality (as lower categories of staff perform functions expected of those in higher categories) and efficiency (higher categories of staff performing functions expected to be done by lower categories) (Daviaud & Chopra 2008). A study by De Wet, Wouters and Engelbrecht (2011) concluded that nurses perform a disproportionate number of tasks that could possibly be completed competently by community health workers, demonstrating an instance where staff members are used inefficiently.

To address these quality and efficiency issues, time and motion studies (TMS) have been used in different parts of the world by continuously and objectively observing healthcare professional’s work whilst also recording the time it takes to complete clinical and non-clinical tasks (Zheng, Guo & Hanaur 2011). Hontelez *et al*. (2012) conducted a TMS in a public healthcare facility in rural KwaZulu-Natal, South Africa to determine the proportion of time that healthcare staff spent on directly related activities (e.g. talking to patients, venipuncture, physical examination and writing), indirect activities (e.g. discussing clinical issues with other healthcare workers and administrative work) and other activities (e.g. breaks and idle time). The authors found that an average workday for a nurse lasted 7.1 hours and they concluded that nurses spent 83% of their time on direct patient activities, 9% on indirect patient activities, and 9% on other activities. The average time a nurse spent with a patient was 10 minutes (95% CI: 6–13 minutes) (Hontelez *et al*. 2012). This is slightly lower than the 12 minute ideal patient consultation time, defined as the (average) time that is recommended by current norms and standards to communicate with the patient about their medical history and ask the relevant questions (Carlsson *et al.* 2013; Meel 2003). Using the information gained from the TMS, Hontelez *et al*. (2012) were able to estimate the number of HIV healthcare workers that would in theory be required to achieve universal access to treatment of HIV in South Africa, thus setting a precedent for the use of the TMS to inform human resource strategies.

Human resource strategies should be more focused on nurses because they are the frontline healthcare professionals in primary healthcare settings in South Africa. Although many nurses are needed for the overburdened healthcare system, the shortage of nurses continues to widen with increasing demand without a commensurate increase in the supply of trained nurses (Mokoka, Oosthuizen & Ehlers 2010). This is in part a result of the provincial healthcare budget cuts that nurses rely on for training finance (Daviaud & Chopra 2008). Consequently, with healthcare resources becoming increasingly limited, the need for innovative strategies to maximise the effectiveness of the care delivered with the existing human resources becomes even more crucial.

Understanding how nurses spend their time is essential to developing innovative ways to improve work processes, organisational culture and workforce management. Therefore, this study sought to ascertain the time that CNPs in fixed clinics and mobile clinics in one district of the Western Cape spend on (curative) core functions and non-core functions.

In this study, we defined curative functions as specialised treatment functions that encompass the CNPs’ job responsibilities, and non-core functions as follow-up activities that may not require the specialised care of a CNP who has gone through a curative training program. In addition, fixed clinics generally have more detailed services, equipment and skills mix, whereas mobile clinics generally aim to address minor ailments in areas where access to healthcare services is limited. Information from this rural district should be of particular use because it offers the opportunity to improve the distribution of nurses within South Africa more equitably. This is important because it prioritises attracting and retaining nurses in rural areas, from where many of the student nurses have migrated (Chen *et al*. 2004). Furthermore, the information gained from examining the CNPs’ time allocation in these facilities can help generate suggestions as to how to increase the productivity and efficiency of rural CNPs through the proper distribution of staff members and specific work activities.

## Methods

### Design

This was a cross-sectional study aimed at describing the work activities that CNPs in a district of the Western Cape performed during an eight-hour workday. It was a descriptive study in the sense that it makes no inferences on the drivers of the measured activities. This proposition is in line with standard definitions of descriptive studies as being ‘concerned with and designed only to describe the existing distribution of variables, without regard to causal or other hypotheses’ (Last 1988).

### Sampling

Purposive sampling was used to select the facilities for the study: in each sub-district, a selection of community day centres, fixed clinics and mobile clinics were included. Facilities were then grouped for sampling according to their: (1) staff complement and composition and resources available – for example, pharmacists who dispense curative medication; professional nurses who deliver promotive and preventative services; and triage staff; and (2) package of services delivered in the facilities, such as a comprehensive care package of services versus only selective services. CNPs with less than two years of practical experience were purposely excluded from participating in the study to allow for nurses who have been working in the facilities to be assessed.

### Data collection method

The same teams visited the sites between August and September 2013. The site visit team consisted of two qualified CNPs who observed the participating CNPs and did timekeeping using an MS Excel electronic data-capturing tool. CNPs conducted the data-capturing because they are the most informed about the proper responsibilities of a CNP, and can expertly detect and distinguish between various CNP activities without needing to interrupt any consultations to time the various activities accurately. The site visit team observed each participating CNP at his or her facility for an entire workday. Facilities were requested to prepare a consulting room for the study. Each consultation with a client was recorded from the time the client entered the consultation room until the time the consultation was concluded. The site visit team also noted all the other tasks performed by the participating CNP. In addition, the MS Excel template was used to categorise the components of each consultation in order to separate curative (core) activities from non-core functions.

## Ethical considerations

Site visits were conducted by CNPs who have previously been nurse trainers, have had many years of experience and were aware of the protocols regarding ethics and patient care. Patients were introduced to the CNP team so that they felt comfortable to ensure that they could communicate freely with the CNP team at any time during the consultation. In addition, the CNP team avoided interfering with the normal patient care routine during the observations. However, in the interests of the patient, they were instructed to interfere whenever the quality of care delivered to the patient seemed to have been compromised in any way during the visit. This was not a problem as these situations were rare.

Having the CNP team observe the clinic CNPs introduced informal training opportunities for the latter since the CNP team could advise them when they faced challenges or had questions. It is therefore unlikely that the presence of the CNP team had a negative effect on patient care – indeed, it is more likely that the patients and the clinic CNPs benefited from their presence because of the opportunities for mentoring and enhanced care. Lastly, the confidentiality of patient information was strictly maintained since no patient interviews were conducted and the data collected did not reveal any confidential patient information.

### Data analysis

The data collected about the time spent was updated with facility and participant information. The data was also consolidated and analysed using STATA version 11.0. The median time spent on the various activities was calculated per observed nurse per day and by facility. The analysis also showed the frequency of the activities and the distinctions between curative/core activities and non-curative/core functions and between fixed clinics and mobile clinics. Time spent on activities and the health facility staff profile were correlated. Regression analysis was also done to identify predictors of the total time a CNP spent with patients. The results were presented using tables and graphs.

## Results

One CNP was observed in each of the 15 selected rural primary healthcare facilities (12 fixed clinics and 3 mobile clinics) in the 5 sub-districts of a district in the Western Cape. For all 15 CNPs observed, the median years of post-basic experience, defined as any extra experience beyond basic nurse training, for CNPs was 21 years (IQR: 13–29 years). The median years for post-curative experience, defined as any extra experience beyond CNP training, was 6 years (IQR: 4–8 years). In these facilities, the nurses saw a total of 530 patients during the observation period, of whom 23.8% were from District 1, 27.6% from District 4, 22.6% from District 5, 19.4% from District 3 and 6.6% from District 2. One-third of the patient visits (33.4%) took place at mobile clinics. The median patient age was 30 (IQR: 9–46 years).

The results are based on 1410 activities measured whilst observing 15 nurses for 134.25 hours. The nurses across the various clinics spent similar amounts of time at work daily, ranging from 8.5 hours to 9.25 hours. The average time spent at work daily was about 9 hours (mobile-clinic nurses: 8 hours 55 minutes; fixed-clinic nurses: 8 hours 57 minutes) (Table 1).

**TABLE 1:** Profile of observed CNPs for one day by healthcare facility.

Sub-district	Facility	Years of experience post basic	Years of experience post curative	Total hours worked	Total number of patients seen	Total nursing staff capacity (all categories)
Sub-district 1	Fixed facility 1	6	3	09:05	27	10
Sub-district 2	Fixed facility 2	11	4	09:00	12	7
Sub-district 3	Fixed facility 3	19	4	09:00	23	7
Sub-district 1	Fixed facility 4	21	9	09:00	19	7
Sub-district 4	Fixed facility 5	29	8	08:27	33	4
Sub-district 5	Fixed facility 6	38	9	08:57	34	3
Sub-district 4	Fixed facility 7	6	4	09:00	35	2
Sub-district 3	Fixed facility 8	19	6	08:56	36	2
Sub-district 5	Fixed facility 9	13	2	09:00	31	3
Sub-district 2	Fixed facility 10	36	4	08:49	23	6
Sub-district 1	Fixed facility 11	28	8	09:15	40	2
Sub-district 4	Fixed facility 12	27	8	08:59	40	3
Sub-district 3	Mobile facility 1	28	2	09:00	44	2
Sub-district 5	Mobile facility 2	18	9	08:30	55	2
Sub-district 4	Mobile facility 3	32	8	09:15	78	2
**Total**	**-**	**-**	**-**	**134:13**	**530**	**-**

The median number of patients seen per day per observed nurse was 34 (IQR: 23–40 patients). This number ranged greatly, from a low of 12 patients per observed nurse per day at a fixed facility to a high of 78 patients per observed nurse per day at a mobile facility. The median number of patients seen per nurse per day was almost twice as high in the mobile clinics (55 patients/day) as at fixed clinics (33 patients/day).

**FIGURE 1:**
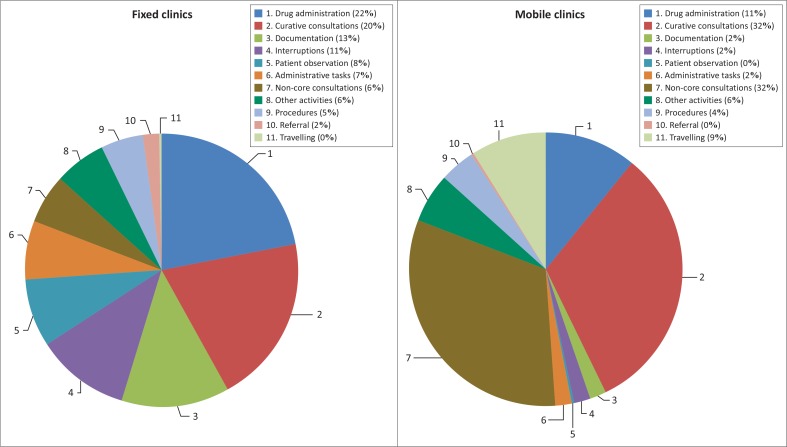
Breakdown of activities.

Figure 1 shows that the most frequently performed activity in fixed clinics is drug dispensing (21.90%) followed by curative consultations (20.28%), documentation of clinical care (12.99%), and interruptions (10.71%). The pattern differed slightly in mobile clinics, with approximately 32% of all measured activities being for curative and another 32% non-core function consultations, followed by drug dispensing (10.55%), travelling (8.89%) and interruptions (1.56%). About 23% of all consultations by CNPs in the fixed clinics were for non-core function consultations. This proportion was higher in mobile clinics with about 50% of all consultations by CNPs being for non-core function consultations. Approximately 42% of all non-core function consultations were for integrated management of childhood illnesses (IMCI) followed by family planning (19.21%) and basic antenatal care (BANC) (15.89%). Table 2 shows a breakdown of the frequency of the non-core functions performed at facility level.

**TABLE 2:** Frequency of specific non-core function consultations in fixed clinics and mobile clinics.

Consultation type	Average frequency for all clinics (%)	Proportion of overall frequency due to fixed clinics (%)	Proportion of overall frequency due to mobile clinics (%)
Basic antenatal care	15.89	83.33	16.67
Well-baby programme	33.11	8.00	92.00
Family planning	19.21	37.93	62.07
Integrated management of childhood illnesses	8.61	84.62	3.17
Tuberculosis drug administration	3.97	66.67	33.33
Mental health follow-up	2.65	75.00	25.00
Pap smear	3.97	100.00	0.00
Post-natal care	2.65	75.00	25.00
Sexually transmitted infection	8.61	76.92	23.08

The total time CNPs spent per patient in fixed clinics (13, IQR: 8–22 minutes) was more than three times greater than the total time CNPs spent per patient in mobile clinics (3, IQR: 2–5 minutes). Table 3 shows the breakdown of the various times spent per activity. This indicates how CNPs in fixed clinics in general spent more time than CNPs in mobile clinics when performing each of the activities measured.

**TABLE 3:** Median time spent per activity.

Activities	Median time (min) spent (IQR)	

	Fixed	Mobile
Curative consultations†	10 (6-15)	3 (2-5)
Non-core consultations‡	12 (6-19)	3 (2-5)
Drug dispensing	2 (1-4)	1 (1-2)
Procedures	2 (1-4)	2 (1-2)
Interruptions§	2 (1-4)	2 (1-5)
Administrative tasks	2 (1-5)	-
Referral	1 (1-2)	2
Documentation	2 (1-4)	1
Physical assessment	2 (1-5)	1
Total time spent per patient	13 (8-22)	3 (2-5)
Other activities¶	7 (2-30)	6 (2-9)

† Curative consultations refers to patient history-taking time for services that are directly included in the CNPs’ job responsibilities

‡ Interruptions refers to breaks during consultations resulting from either phone calls, someone else coming into the patient room, or for reasons unrelated to the care of the particular patient

§ Non-core consultations refers to patient history-taking time for services that are not directly included in the CNPs’ job responsibilities

¶ Other activities refers to any breaks and other miscellaneous activities.

The analysis shows that the more time nurses spend with the patient, the more time they also spend on procedures (*r* = 0.55) and dispensing drugs (*r* = 0.40). The Pearson correlation analysis also showed that there was a direct relationship between the total time CNPs spend with patients and the number of professional nurses (*r* = 0.37), number of CNPs (*r* = 0.39) and the total number of nursing staff at a health facility (*r* = 0.40). This means that the more nurses there are in a facility, the more time CNPs spend on consultation.

The regression analysis showed that for every one unit increase in total staff, the total time CNPs spend with a patient will increase by 2.40 minutes (*p* = 0.00), holding everything else constant. In agreement with an earlier result in this report, as patients move from mobile clinics to fixed clinics, the total time the CNPs spent with them increases by 11.56 minutes (*p* < 0.00). The regression analysis also showed that with every additional enrolled nurse in a clinic, the CNP spends about 4.27 minutes less with a patient. Years of experience was found to be inversely related to the total time spent with patients. In other words, the more experienced a CNP was, the less time they spent with a patient. In all, about 30% of the variation in the total time a CNP spends with each patient is determined by the type of facility (fixed or mobile), the total number of nursing staff in a facility, the number of enrolled nurses in the facility and the years of experience of the CNP.

## Discussion

The results showed that the CNPs in mobile clinics spent below the minimum consultation time recommended by most research studies and professional standards. Although it is possible to infer that the reduced consultation time could be indicative of the high productivity of the CNPs in the mobile clinics, studies have supported the idea that reducing the amount of time dedicated to patient consultations negatively affects patient satisfaction and the quality of care delivered (Dugdale, Epstein & Pantilat 1999; Gallagher *et al.* 2005; Morrell *et al.* 1986; Ridsdale *et al*. 1989). As a consequence of shorter visits, it was found that healthcare professionals have less time to address patient needs and patients have less time to communicate their problems. This lack of proper communication between the healthcare provider and the patient can invariably affect health outcomes since spending enough time on patient visits results in higher adherence rates, fewer medical errors, and thus improved health outcomes (Blendon *et al*. 2002; Zolnierek & DiMatteo 2009). For instance, Nachega *et al*. (2012) found that enhanced patient–provider communication is crucial to improving ART adherence in HIV patients. It may, therefore, be more likely that the shorter consultation times challenges the quality and effectiveness of the services mobile clinics provide, as well as their capacities to deal with certain health concerns that may require more consultation time.

The study findings also indicated that CNPs in fixed clinics generally spent about the minimum consultation time recommended by most research studies and professional standards. It is possible that longer consultation time may imply a greater quality of care delivered and a positive impact on patient satisfaction as a result of enhanced communication between the CNPs and patients (Dugdale *et al.* 1999; Gallagher *et al.* 2005; Morrell *et al.* 1986; Ridsdale *et al.* 1989). However, whether or not the nurses in the fixed clinics used their longer consultation time efficiently without compromising other duties is still unknown. Moreover, based on these findings, further investigation is needed to ensure that the mobile clinics and fixed clinics are delivering effective, efficient and high-quality services to their patients.

The results also indicated that nurses at fixed clinics were slower at performing non-core functions compared to curative functions, which may be representative of the CNPs’ expertise in performing skilled tasks. This further supports the idea that CNPs are more efficient at their prescribed roles. Thus, it could be more efficient for the clinic to task shift some of the non-core functions to other supportive, less skilled healthcare staff members in the clinic so that the CNPs can focus on their core curative duties. Not only has task shifting been noted for cost savings with regard to healthcare worker training costs, but studies have also shown that it does not reduce the quality of care delivered. Therefore, this study suggests that task-shifting some of the non-core tasks, in the fixed clinics especially, would be a promising human resource management strategy as it maximises the potential of the available healthcare professionals (Morris *et al*. 2009).

The results showed that well-baby consultations, family planning consultations and basic antenatal care consultations were the most popular types of non-core consultation across all the clinics examined. Therefore, if the facility managers wanted to task shift some non-core consultations to lower-skilled healthcare workers, the managers could use the collected TMS information, such as the data in Table 2, to determine which tasks should be prioritised when deciding on tasks to assign to the other healthcare workers. However, it should be noted that these decisions should be made whilst considering the limitations that less skilled workers have regarding medical expertise (Morris *et al*. 2009).

The study indicated that clinics with a higher workload based on patient headcount and staff complement spent less time overall on consultations compared to those with lower workloads. This resonates with studies which have found that increased workload and staff shortage reduces the amount of time available for patient care activities, and thus compromises patient health outcomes (Duffield *et al*. 2011; Hendrich *et al.* 2008). This suggests that CNPs from clinics with smaller workloads based on patient headcount and staff complement could deploy some of their nurses to other clinics with higher workloads in order to use fully the skills of scarce CNPs in the field, assuming the smaller workload clinics maintain a sufficient number of nurses to meet their service demands.

The study’s findings show that the distribution of CNPs per patient headcount across each sub-district is uneven and thus elicits the conclusion that there is an even greater need than previously thought to use an appropriate staffing calculation to place CNPs at facilities. Since CNPs and nurses in general are critical, scarce and costly human resources, such calculations should also take into consideration the different categories of nurses and the relative demand for each of their skillsets and/or competencies for more cost-effective decision-making. An appropriate model for nurse allocation per facility should be employed to ensure a better distribution of core and non-core skillsets across facilities in the district, to maximise the potential of the available CNPs whilst increasing access to healthcare services (Daviaud & Chopra 2008; Fulton *et al*. 2011).

Furthermore, it has been shown that having the proper density of healthcare workers in a given area can have a great impact on the effectiveness of healthcare interventions (Chen *et al*. 2004). Thus, redistributing CNPs so that sub-districts and districts have the minimum density of nurses could maximise the efficiency and productivity of nurses, which is essential to the promotion of quality care (Chen *et al*. 2004; Hendrich *et al.* 2008). Whilst this is the ideal, it may not be practical since the current CNPs may not want to relocate from their current locations. Therefore, healthcare worker density and distribution patterns could be taken into account more earnestly when first deploying CNPs to facilities after graduation in order to improve the present inequitable distribution of CNPs.

As mentioned previously, the study found that there was a 2.4-minute increase in total patient time for every one unit increase in staff. Information such as this could be used by the facilities’ management to calculate the appropriate number of staff members for their facility that would move their present patient consultation time towards the accepted standard patient time of 12 minutes per consultation. Moreover, in particular, this type of information could have important implications for the mobile clinics, which serves a vulnerable population as their patients do not have regular access to healthcare facilities. Adding the appropriate number of staff to these mobile healthcare facilities would go a long way to better addressing the needs of this population sooner. This could prevent health conditions from getting progressively worse and decrease the number of patients who would need to use more specialised facilities (e.g. the emergency room) for more serious conditions (Alexy & Elnitsky 1998). Adding an extra nurse to the skills mix of the mobile clinics could therefore enhance the long-term health of these rural communities.

## Study limitations

This study has several limitations. First, the analysed data was based on a small sample size of CNPs (*n* = 15) with only one CNP per facility, 2–4 facilities per sub-district, and a disproportionate number of fixed clinics (*n* = 12) to mobile clinics (*n* = 3). The small sample of CNPs may therefore not be representative of the facilities examined. In addition to the disproportionate type and number of facilities per sub-district overall, this is not sufficient to show the true situation in the sub-districts that were studied. Although the sample size is small, though, general inferences can still be developed from the sample since purposive sampling was used to select the facilities targeted for this study.

Secondly, as mentioned in the introduction, this was a cross-sectional study and because the data was only gathered at one point in time, we cannot establish temporality or determine causation. However, we may still use our findings to generate inferences and hypotheses for further investigation of what is an appropriate skills mix for delivering quality care in these rural fixed and mobile clinics.

Thirdly, the CNPs were aware that they were being observed by two other CNPs. This could have placed added pressure on the observed CNPs to increase their productivity on the day they were studied compared to days when there were no observers.

Fourthly, the activities studied on the one day of observation may not include all of those that CNPs participate in. For instance, they may also participate in less frequent activities such as attending workshops or other training sessions. However, this should not have had a significant effect on the examination of their allocation of time to various daily activities, since these extra, less frequent activities are likely to take up only a small proportion of the total work a CNP puts in each year (Hontelez *et al*. 2012).

Lastly, each of the fixed clinics and mobile clinics studied could have differed by various factors that have been shown to be associated with the productivity of the healthcare workers. For instance, in terms of healthcare worker factors, the CNPs’ productivity can also be affected by their knowledge and skills (e.g. perceptions of patient needs, comprehension of work responsibilities), their health (e.g. compassion fatigue, burnout), and their motivation to fulfil their duties (e.g. affected by their past experiences, personal goals, self-efficacy to carry out their responsibilities). In terms of each facility’s patient population factors, the CNPs’ performance may be influenced by the severity of their patients’ illnesses or the patients’ acceptance of the CNPs’ information. Regarding the environment of the health facility itself, the CNPs’ productivity may differ based on the attitudes of co-workers, the availability of equipment and supplies, the level of supervision, the organisation of patient flow, the presence or lack of additional healthcare workers, and much more (Rowe *et al.* 2005).

This study was conducted on the assumption that the facilities had CNPs who were equally motivated, supported and skilled whilst the study team was examining how patient caseload in particular affected CNP’s productivity and the implications of that for facility skills mix and health worker deployment. Whilst this study specifically focused on the patient caseload factor, more studies are needed to study the effects of the other variables on CNPs’ performance.

## Conclusion

With a shortage of healthcare professionals within an overburdened healthcare system in South Africa, innovative strategies are necessary to catalyse health improvements in the country. This is especially crucial in the rural areas, where access to healthcare can be scarce and irregular. Whilst this study has limitations with regard to its methodology, it nonetheless gives insight into the amount of time CNPs in rural fixed and mobile clinics spend with their patients, and how patient caseload may have an impact on consultation times. The findings also warrant additional studies on how to ensure facilities such as these have the appropriate number and type of healthcare workers who can deliver an effective, efficient and quality service to their patients. Based on these insights, we suggest that task-shifting and the redistribution of staff strategies within sub-districts and districts allow the facilities to better address the medical needs of their patients whilst fully maximising the potential of the existing nurses in a resource-limited setting. This takes into account the counter-balancing effect of the ratio of CNPs to enrolled nurses on the demand for CNPs’ work time. We also propose that the above ideas should be prioritised for task shifting.

Addressing facility staffing and skills mix has great potential for using the full potential of rural healthcare workers whilst enabling them to maintain the quality of services delivered to their patients. Moreover, in accordance to Chen *et al.*’s (2004) model for managing for performance, optimising the staffing and skills mix may also increase coverage of the patient population and enhance the motivation of the healthcare workers, especially those who have high patient caseloads. This, in turn, could lead to more equitable access to healthcare for patients and more efficient and effective health services delivered, and also enhance the quality of the healthcare administered and the responsiveness of the healthcare system to the patients’ needs, all culminating in improved the health for the population (Chen *et al*. 2004).

Task-shifting and adjustments to healthcare worker deployment are two promising strategies with which to tackle staffing and skills mix in these facilities in order to enhance the long-term health of these rural communities. Additional studies are needed to further explore the effect of staffing and skills mix on health outcomes, and how this change in health worker distribution and task shifting may contribute to improved health services.

## References

[CIT0001] Alexy B.B. & Elnitsky C., 1998, ‘Rural mobile health unit: Outcomes’, *Public Health Nursing* 15(1), 3–11. PMID: , 10.1111/j.1525-1446.1998.tb00314.x 9503947

[CIT0002] Blendon R.J., DesRoches C.M., Brodie M., Benson J.M., Rosen A.B., Schneider E. et al., 2002, ‘Views of practicing physicians and the public on medical errors’, *New England Journal of Medicine* 347(24), 1933–1940. PMID: , 10.1056/NEJMsa022151 12477944

[CIT0003] Callaghan M., Ford N. & Schneider H., 2010, ‘A systematic review of task-shifting for HIV treatment and care in Africa’, *Human Resources for Health* 8, 8–16. PMID: , 10.1186/1478-4491-8-8 20356363 PMC2873343

[CIT0004] Carlsson E., Pettersson M., Hyden L.C., Ohlen J. & Friberg F., 2013, ‘Structure and content in consultations with patients undergoing surgery for colorectal cancer’, *European Journal of Oncology Nursing* 17(2013), 820–826. PMID: , 10.1016/j.ejon.2013.07.002 24012188

[CIT0005] Chen L., Evans T., Anand S., Boufford J.I., Brown H., Chowdhury M. et al., 2004, ‘Human resources for health: Overcoming the crisis’, *Lancet* 364(9449), 1984–1990. PMID: , 10.1016/S0140-6736(04)17482-5 15567015

[CIT0006] Coovadia H., Jewkes R., Barron P., Sanders D. & McIntyre D., 2009, ‘The health and health system of South Africa: Historical roots of current public health challenges’, *Lancet* 374(9692), 817–834. PMID: , 10.1016/S0140-6736(09)60951-X 19709728

[CIT0007] Daviaud E. & Chopra M., 2008, ‘How much is not enough? Human resources requirements for primary health care: A case study from South Africa’, *Bulletin of the World Health Organization* 86(1), 46–51. PMID: , 10.2471/blt.07.042283 18235889 PMC2647342

[CIT0008] De Wet K., Wouters E. & Engelbrecht M., 2011, ‘Exploring task-shifting practices in antiretroviral treatment facilities in the Free State Province, South Africa’, *Journal of Public Health Policy* 32, S94–S101. PMID: , 10.1057/jphp.2011.30 21730997

[CIT0009] Duffield C., Diers D., O’Brien-Pallas L., Aisbett C., Roche M., King M. et al., 2011, ‘Nursing staffing, nursing workload, the work environment and patient outcomes’, *Applied Nursing Research* 24(4), 244–255. PMID: , 10.1016/j.apnr.2009.12.004 20974086

[CIT0010] Dugdale D.C., Epstein R. & Pantilat S.Z, 1999, ‘Time and the patient–physician relationship’, *Journal of General Internal Medicine* 14(S1), 34–40. 10.1046/j.1525-1497.1999.00263.x PMC1496869 9933493

[CIT0011] Fulton B.D., Scheffler R.M., Sparkes S.P., Auh E.Y., Vujicic M. & Soucat A., 2011, ‘Health workforce skill mix and task shifting in low income countries: A review of recent evidence’, *Human Resources in Health* 9(1), 1. PMID: , 10.1186/1478-4491-9-1 21223546 PMC3027093

[CIT0012] Gallagher T.J., Hartung P.J., Gerzina H., Gregory S.W. Jr & Merolla D., 2005, ‘Further analysis of a doctor–patient nonverbal communication instrument’, *Patient Education and Counseling* 57(3), 262–271. PMID: , 10.1016/j.pec.2004.06.008 15893207

[CIT0013] Hendrich A., Chow M.P., Skierczynski B.A. & Lu Z., 2008, ‘A 36-hospital time and motion study: How do medical-surgical nurses spend their time?’, *The Permanente Journal* 12(3), 25. PMID: . 21331207 10.7812/tpp/08-021 PMC3037121

[CIT0014] HERD Health Economics and HIV/AIDS Research Division, 2009, Human resources for health: A needs and gaps analysis of HRH in South Africa, viewed 4 October 2014, from http://www.heard.org.za/downloads/human-resources-for-health--a-needs-and-gaps-analysis-of-hrh-in-south-africa.pdf

[CIT0015] Hontelez J.A., Newell M.L., Bland R.M., Munnelly K., Lessells R.J. & Bärnighausen T., 2012, ‘Human resources needs for universal access to antiretroviral therapy in South Africa: A time and motion study’, *Human Resources in Health* 10(1), 39. PMID: , 10.1186/1478-4491-10-39 23110724 PMC3529683

[CIT0016] Last J.M, 1988, *A dictionary of epidemiology,* 4th edn., Oxford University Press, New York, Oxford, Toronto.

[CIT0017] Meel B.L., 2003, ‘Adequacy and efficiency of nursing staff in a child-welfare clinic at Umtata General Hospital, South Africa’, *African Health Sciences* 3(3), 127–129. PMID: . 14676718 PMC2141607

[CIT0018] Mokoka E., Oosthuizen M.J. & Ehlers V.J, 2010, ‘Retaining professional nurses in South Africa: Nurse managers’ perspectives‘, *Health SA Gesondheid* 15(1), 1–9. 10.4102/hsag.v15i1.484

[CIT0019] Morrell D.C., Evans M.E., Morris R.W. & Roland M.O., 1986, ‘The “five minute” consultation: Effect of time constraint on clinical content and patient satisfaction’, *British Medical Journal (Clinical Research edn.)* 292(6524), 870–873. PMID: , 10.1136/BMJ.292.6524.870 3083919 PMC1339978

[CIT0020] Morris M.B., Chapula B.T., Chi B.H., Mwango A., Chi H.F., Mwanza J. et al., 2009, ‘Use of task-shifting to rapidly scale-up HIV treatment services: Experiences from Lusaka, Zambia’, *BMC Health Services Research* 9(1), 5. PMID: , 10.1186/1472-6963-9-5 19134202 PMC2628658

[CIT0021] Nachega J.B., Morroni C., Zuniga J.M., Schechter M., Rockstroh J., Solomon S. et al., 2012, ‘HIV treatment adherence, patient health literacy, and health care provider-patient communication: Results from the 2010 AIDS treatment for life international survey’, *Journal of the International Association of Physicians in AIDS Care (JIAPAC)* 11(2), 128–133. PMID: , 10.1177/1545109712437244 22361449

[CIT0022] Ridsdale L., Carruthers M., Morris R. & Ridsdale J., 1989, ‘Study of the effect of time availability on the consultation’, *Journal of the Royal College of General Practitioners* 39(329), 488–491. PMID: . 2558202 PMC1712205

[CIT0023] Rowe A.K., de Savigny D., Lanata C.F. & Victora C.G., 2005, ‘How can we achieve and maintain high-quality performance of health workers in low-resource settings?’, *Lancet* 366(9490), 1026–1035. PMID: , 10.1016/S0140-6736(05)67028-6 16168785

[CIT0024] Zheng K., Guo M.H. & Hanauer D.A., 2011, ‘Using the time and motion method to study clinical work processes and workflow: Methodological inconsistencies and a call for standardized research’, *Journal of the American Medical Informatics Association* 18(5), 704–710. PMID: , 10.1136/amiajnl-2011-000083 21527407 PMC3168304

[CIT0025] Zolnierek K.B.H. & DiMatteo M.R., 2009, ‘Physician communication and patient adherence to treatment: A meta-analysis’, *Medical Care* 47(8), 826. PMID: , 10.1097/MLR.0b013e31819a5acc 19584762 PMC2728700

